# Quorum-sensing and cheating in bacterial biofilms

**DOI:** 10.1098/rspb.2012.1976

**Published:** 2012-10-03

**Authors:** Roman Popat, Shanika A. Crusz, Marco Messina, Paul Williams, Stuart A. West, Stephen P. Diggle

**Affiliations:** 1 School of Molecular Medical Sciences, Centre for Biomolecular Sciences, University of Nottingham, University Park, Nottingham NG7 2RD, UK; 2 Department of Zoology, University of Oxford, The Tinbergen Building, South Parks Road, Oxford OX1 3PS, UK

**Keywords:** quorum-sensing, biofilms, public goods, spatial structure, cooperation, cheating

## Abstract

The idea from human societies that self-interest can lead to a breakdown of cooperation at the group level is sometimes termed the public goods dilemma. We tested this idea in the opportunistic bacterial pathogen, *Pseudomonas aeruginosa*, by examining the influence of putative cheats that do not cooperate via cell-to-cell signalling (quorum-sensing, QS). We found that: (i) QS cheating occurs in biofilm populations owing to exploitation of QS-regulated public goods; (ii) the thickness and density of biofilms was reduced by the presence of non-cooperative cheats; (iii) population growth was reduced by the presence of cheats, and this reduction was greater in biofilms than in planktonic populations; (iv) the susceptibility of biofilms to antibiotics was increased by the presence of cheats; and (v) coercing cooperator cells to increase their level of cooperation decreases the extent to which the presence of cheats reduces population productivity. Our results provide clear support that conflict over public goods reduces population fitness in bacterial biofilms, and that this effect is greater than in planktonic populations. Finally, we discuss the clinical implications that arise from altering the susceptibility to antibiotics.

## 1. Introduction

The growth and success of bacterial populations depends upon the production of extracellular factors that are secreted to perform many functions such as nutrient acquisition, protection from the environment and the creation of enemy-free space [1,2]. These factors provide a benefit to the local population of cells, and so their production is potentially vulnerable to exploitation by ‘cheats’ that avoid the cost of producing them, while benefiting from those produced by others. This problem, sometimes referred to as the ‘public goods dilemma’, is general to all biological populations where cooperation enhances population fitness, because while a group of individuals would benefit from cooperation, cooperation may not be stable if individuals can gain from pursuing their own selfish interests [3,4]. A clear prediction is that measures of group success, such as growth or productivity, will be negatively correlated with the proportion of cheats, as has been found in a number of studies with bacteria and other microbes [5–12].

However, previous work on cooperation and cheating in bacteria has mainly focused on cells growing in planktonic liquid cultures. The relevance of such studies may be limited by the fact that the vast majority of natural bacterial populations are thought instead to exist as slimy, well-structured multicellular communities, termed biofilms [13,14]. Biofilms are relevant to clinical environments because they are thought to play a key role in the ability of organisms to tolerate antibiotics and persist in long-term chronic infections [15–17]. It is likely that biofilms change the nature of social interactions between cells, because they lead to structured and densely packed populations, with considerable potential for both competition and cooperation between cells [18]. There are a number of reasons why this could either increase or decrease the consequences of cheating, for example by facilitating the sharing of public goods, or by keeping cheats and cooperators segregated [19–27].

Here we examine the fitness consequences of cheating at the population level, and test whether this varies between planktonic and biofilm populations. We use the bacterium *Pseudomonas aeruginosa*, an opportunistic, multi-antibiotic-resistant pathogen of plants and animals (including humans) [28]. Biofilms are thought to play a key role in the ability of this species to tolerate antibiotics and survive within long-term chronic infections in the lungs of humans with cystic fibrosis, where it is an important cause of morbidity and mortality [15]. In this species, the production of many extracellular factors, including those involved in biofilm formation, are controlled by quorum-sensing (QS). QS is the process by which small diffusible signalling molecules are released, accumulate, and when they reach a sufficient concentration, drive the expression of genes coding for extracellular factors [29,30]. Previous studies, both *in vitro* and *in vivo*, have shown that the production of these extracellular factors is a cooperative trait that provides a benefit to the local population of cells [9,10,31–35].

We used a wild-type (WT) *P. aeruginosa* strain (PA01) with a fully functioning QS system as our cooperator, and an isogenic *lasR* mutant that does not respond to signal as our putative cheat. In our study, the term cheat implies that the cell cannot use a specific nutrient source but can exploit the extracellular digestion of that resource by other cells (cooperators) in the population [9,34]. Our specific aims are to test whether: (i) QS cheating occurs in biofilms; (ii) the thickness and density of biofilms is influenced by QS cheats; (iii) the relationship between productivity and percentage QS cheats differs between planktonic and biofilm populations; (iv) the susceptibility of biofilms to antibiotics varies with the percentage of cheats; and (v) coercing cooperating cells to increase their level of cooperation influences the effect of cheats on population productivity.

## 2. Methods

### (a) Bacterial strains and growth media

The strains we used in this study were the *P. aeruginosa* WT strain PAO1 and an isogenic insertion mutant in the QS regulator *lasR* (PAO1 *lasR*::Gm). To determine QS activity during growth, we fused the promoter of *lasB* to the *luxCDABE* operon in a mini-CTX*lux* delivery system to have the fusion integrated in single copy in the chromosome [36]. To grow the strains, we used a rich medium, Lysogeny Broth (LB), and a defined QS medium (QSM). QS is required for maximal growth in QSM, owing to the requirement for the production of QS-dependent extra-cellular proteases. QSM consisted of aqueous M9 minimal salts solution and supplemented with filter-sterilized carbon sources bovine serum albumin (1% w/v) and casamino acids (CAA 0.1% w/v) [9,34]. For growth assays, we first inoculated pre-cultures into 25 ml universal tubes containing 6 ml of LB medium using a single colony, and incubated at 37°C for 18 h, with shaking. We then washed the pre-cultures in sterile QSM twice and corrected to optical density_600_ (OD_600_) 1.0 before inoculating into subsequent experiments at an initial OD_600_ of 0.01.

### (b) Growth and measurement of bacterial biofilms using flow cells

We cultured flow-cell biofilms using a modified flow-cell apparatus, the setup of which is described in detail elsewhere [37]. Although we were interested in the summed growth of mixed biofilms, it was also possible to visually differentiate between PAO1 and the *lasR* mutant, using confocal microscopy. To achieve this, we labelled both PAO1 and PAO1 *lasR*::Gm with plasmids pMMG and pMMR, expressing Green Fluorescent Protein (GFP) and mCherry, respectively. Vectors pMMG and pMMR are derived from the expression vector pME6032 [38], in which *lacI* was deleted to obtain constitutive gene expression. We inoculated flow chambers by injecting 200 µl of pre-culture and then incubated these statically at 37°C for 1 h to allow for cell attachment. We then pumped a 1 : 10 dilution of QSM through the flow chambers at 50 µm s^−1^ while maintaining the temperature at 37°C and allowed the experiment to run for 7 days. Following incubation, we visualized biofilm growth using a Zeiss LSM 510 UV META Kombi confocal system on an inverted Zeiss Axiovert 100 M microscope (Carl Zeiss, Germany). We used a 488 nm laser coupled with the 505–530 band pass filter to detect GFP and a 543 nm laser coupled with the LP560 filter to detect mCherry. We calculated biofilm parameters using Comstat [39], including biovolume (µm^3^/µm^2^ reported as micrometres), average thickness and maximum thickness (both in micrometres). We used three flow channels containing either PAO1 WT, PAO1 *lasR*::Gm or a 1 : 1 mixture of both, and we collected six, six and three replicate confocal *z* stacks, respectively. We created the 1 : 1 mixture by mixing equal volumes of cultures corrected to OD_600_ 1.0.

### (c) Growth and competition in a bead biofilm system

To determine whether a *lasR* mutant could exploit the PAO1 WT in a biofilm, we employed a submerged bead biofilm culture system [40]. In this experiment, we submerged a plastic bead in 4 ml QSM contained in a 30 ml universal tube, with shaking at 50 r.p.m. and incubated that at 37°C for 48 h. We washed the beads four times by removing each time the liquid phase and adding 6 ml of fresh QSM medium. We then stripped the biofilms from the bead by vortexing for 1 min in 3 ml fresh medium. We plated biofilm cells out to single colonies and determined the relative frequency of PA01 WT and PAO1 *lasR*::Gm (carrying a promoterless mini-CTX*lux* chromosomal insertion). As a control, to ensure we had separated biofilm and liquid phases, we plated out the final wash step and determined the density of planktonic cells remaining in the wash (3.02 10^7^ CFU ml^−1^ ± 6.46 × 10^6^ s.e. mean) and the density of the cells in the resuspended biofilm (3.60 × 10^12^ CFU ml^−1^ ± 6.79 × 10^11^ s.e. mean).

To determine whether the PAO1 *lasR*::Gm mutant was able to exploit the WT, we initiated 10 mixed cultures at an initial PAO1 *lasR*::Gm frequency of 0.044 ± 0.0073 (s.e. mean). We calculated the relative fitness of the mutant using the formula *w* = *p*1(1−*p*_0_)/*p*_0_(1−*p*_1_), where *p*_0_ and *p*_1_ are the proportion of *lasR* mutants in the population before and after incubation, respectively [11]. If over the time of the experiment, the growth rate of the mutant was twice that of the WT, its relative fitness would be 2. This calculation does not account for differences in overall population growth rate between treatments; however, the final densities of resuspended biofilm (as estimated by CFU ml^−1^) did not differ significantly (*t*_14.6_ = 1.2, *p* = 0.247). To determine whether cheating in biofilms was mediated through the production of extracellular factors (specifically proteases), we experimentally added purified elastase (porcine elastase) directly to the growth medium. We added 0.0067 units of elastase per millilitre [34] to 10 replicate cultures containing beads.

### (d) Cheat frequency, productivity and antibiotic resistance in microtitre plates

To determine biofilm growth in microtitre plates, we inoculated strains in 200 µl QSM in polypropylene flat bottom 96 well plates (Greiner) at an initial starting OD_600_ of 0.01. After incubation, we washed each well in sterile phosphate buffered saline buffer twice and fixed for 30 min in 100 per cent methanol. We then air-dried the wells and stained with 0.1% Crystal Violet (CV) for 15 min. After staining, we washed the wells three times in distilled water. We then resolubilized the CV using 33 per cent glacial acetic acid and measured OD_595_ using a Tecan Infinite 200 microplate reader. CV staining has been shown to correlate well with several other biofilm quantification assays [41,42]. To determine the effect of cheat frequency on productivity in both liquid cultures and biofilms, we inoculated 200 µl QSM in 96 well plates at an initial OD_600_ of 0.01, containing varying mixtures of cooperator (PAO1) and cheat (PAO1 *lasR*::Gm). To determine planktonic growth, we monitored OD_600_ for 24 h at 37°C in a Tecan microplate reader. In separate parallel experiments, to determine biofilm growth, we stained the 96 well plate with CV as described earlier. When we did this, we observed that the biofilm had formed a ring around the top of the well at the air–liquid interface. Our measurements of OD, which occur vertically through the well, are therefore not obscured by the formation of a biofilm. We measured productivity in both liquid and attached communities and in effect, estimated total productivity of each well. In order to compare planktonic and biofilm productivity, we analysed productivity as a proportion of the equivalent culture in the absence of cheats. To challenge biofilms with tobramycin (Tb), we removed the medium from the wells and replaced it with sterile QSM containing 60 mg l^−1^ Tb and incubated for a further 3 h. As a control, we added sterile medium instead of Tb. We then determined biofilm mass by CV staining. We calculated the proportional biofilm remaining after (Tb) treatment using the following equation: Biofilm Remaining = CV stain (at mix frequency i) with Tb/CV stain (at mix frequency i) without Tb. Our estimates of biofilm remaining after Tb treatment are likely to underestimate the viable cell number within the biofilms as Tb lyses cells.

### (e) Determination of productivity and quorum sensing activity with exogenous signal molecule

To measure QS activity under cheat load, we used PAO1 and PAO1 *lasR*::Gm transformed with a *lasB*::*lux* reporter, because *lasB* is a good indicator of *lasIR*-mediated QS activity [43]. We inoculated three replicate experiments of different starting percentages of cheat (1, 10, 25, 50 and 100%) in 200 µl liquid QSM cultures in the presence and absence of 50 µM *N*-(3-oxododecanoyl)-l-homoserine lactone (3-oxo-C12-HSL). We monitored growth (OD_600_) and luminescence (relative light units, RLU) over 24 h in a Tecan microplate reader. We analysed growth at 24 h (productivity) and luminescence (QS activity) corrected for culture density (RLU/OD_600_) at the peak of *lasB* expression at 11 h.

### (f) Statistical analyses

We performed all statistical analyses and data visualizations using the open source statistical platform R [44], using the packages included in R v. 2.14.2. We compared monoculture and co-culture flow-cell biofilm biomass density using two-way ANCOVA and a log transformation. We compared the relative fitness of PAO1 *lasR*::Gm in bead biofilms, using welch one and two sample *t*-tests with unequal variances. We analysed flow-cell biofilm architecture by ordered heterogeneity (OH) combining ANOVA F statistics with Spearmans rank correlation [45]; planktonic and biofilm productivity in monocultures and mixed cultures using ANCOVA with a two-way interaction between percentage cheat and growth mode and a log–log transformation; biofilm survival by linear modelling and evaluated model coefficients with ANOVA. We analysed mixed liquid culture density and *lasB* expression by linear modelling and ANOVA with a log transformation. We checked the assumptions of ANOVA for each analysis where ANOVA was used. All data have been uploaded into the Dryad Digital Repository.

## 3. Results

### (a) *lasR* mutants act as social cheats in biofilms

**Figure 1.**
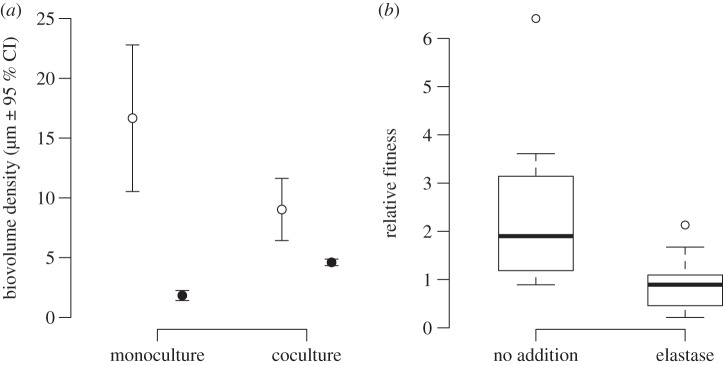
A *lasR* mutant behaves as a social cheat in structured populations. (*a*) When grown in monoculture flow cells, the *lasR* mutant (filled circles) shows reduced growth in QS essential media (QSM). This growth is enhanced by the presence of a WT (open circles) in co-culture. Points represent the mean, and error bars represent the 95% CI around the mean. (*b*) When grown in mixed culture bead biofilms with the WT in QSM, the relative fitness of the *lasR* mutant is higher than the WT (values greater than 1) but this difference in fitness is reduced when synthetic elastase is added to the culture. The open circles represent data points that fall outside 1.5 times the interquartile range.

Previously, it has been shown that *lasR* mutants act as social cheats in well-mixed planktonic cultures [9,10,33]. To test whether a *lasR* mutant behaves as a social cheat in structured biofilms, we used two approaches. First, we examined the relative growth of the PAO1 WT and a *lasR* mutant, when grown in either monoculture or mixed co-culture biofilms in flow cells. In monoculture flow cells, the WT forms biofilms of a greater biomass than the *lasR* mutant (*F*_3,14_ = 84.32, *p* < 0.001; figure 1*a*). In co-culture, the biomass of the WT is reduced, but the biomass of the *lasR* mutant is increased (*F*_3,14_ = 15.45, *p* = 0.0015; figure 1*a*).

Second, we grew biofilms on plastic beads using mixed populations with a starting frequency of the *lasR* mutant of 5 per cent and we then measured the relative fitness of the mutant after a period of growth. To determine whether any change in frequency was owing to the exploitation of WT protease production, we added synthetic elastase to some of the cultures. We found that in co-culture, the *lasR* mutant had a relative fitness of greater than 1 (one sample *t*-test, alternative hypothesis: *μ* ≠ 1, *t*_9_ = 2.73, *p* = 0.023; figure 1*b*) and that this was significantly decreased by the addition of elastase to the culture (two sample welch *t*-test, alternative *μ* ≠ 0, *t*_11.4_ = 2.70, *p* = 0.020; figure 1*b*).

### (b) Cheats decrease biofilm productivity and increase susceptibility to antibiotics

**Figure 2.**
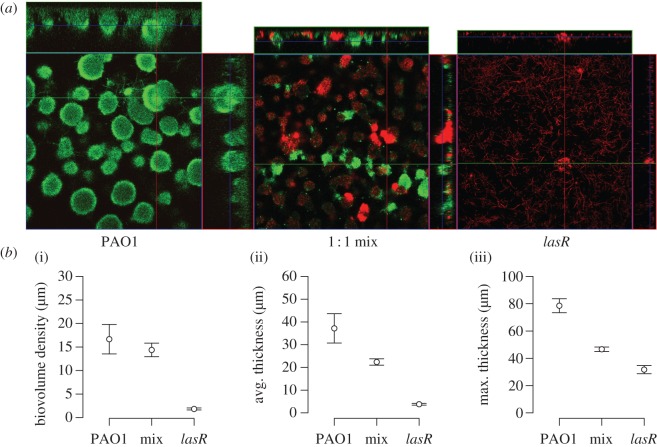
Public goods conflict and biofilm productivity. Biofilms of the PAO1 (cooperator) WT (green), a *lasR* mutant (red) that does not respond to signal or a 50 : 50 mixture were grown in QSM for 7 days in flow chambers, measured with confocal laser scanning microscopy and analysed using Comstat. Both (*a*) representative confocal images and (*b*) Comstat quantifications of (i) biomass, (ii) average thickness and (iii) maximum thickness show that PAO1 forms denser and thicker biofilms than a *lasR* mutant growing in monoculture and a 50 : 50 mixture of the two. Points represent the mean, and error bars represent the s.e. of the mean.

**Figure 3.**
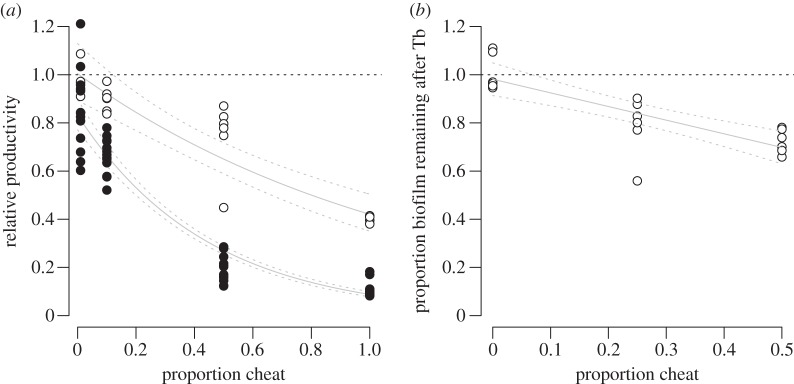
The public goods dilemma, biofilms and antibiotic susceptibility. (*a*) The productivity of populations decreases with increasing cheat frequency but more so in biofilms (filled circles) compared with planktonic cultures (open circles). Values were calculated as a proportion of an equivalent pure WT culture and this is represented by the dashed line at 1. The experiment included six (planktonic) and 12 (biofilm) independent replicates at all values of proportion cheat. (*b*) The proportion of biofilm surviving after exposure to 60 mg l^−1^ tobramycin (Tb) decreases with increasing percentage of cheats in the population. The dashed line at 1 represents the level at which Tb has no effect on the proportion of biofilm surviving. The solid lines represent the results of linear modelling, and the dashed lines represent the 95% confidence limits placed on the linear model.

We then tested how QS cheats influenced the growth of biofilms in QSM using a flow-cell system. Biofilms grown in flow cells begin as sparse attached cells, separated by empty space on the glass coverslip. They then grow in the absence of non-attached cells and in doing so, form the structures seen in figure 2*a*. We found a progressive decrease in biomass density, average thickness and maximum thickness between a WT, a 50 : 50 mixed population and a *lasR* mutant (OH tests; r_S_P_C_ = 0.81, 0.86, 0.93, respectively, and *p* < 0.05 in all three cases; figure 2*a,b*). We found a similar pattern in a microtitre plate biofilm system, with a higher proportion of cheats in the population leading to lower levels of productivity in both planktonic and biofilm cultures (*F*_3,76_ = 328.6, *p* < 0.0001; figure 3*a*). Furthermore, this negative effect of cheats was greater in biofilms, especially at high cheat frequencies (*F*_3,76_ = 12.4, *p* < 0.001; figure 3*a*).

As QS has been shown to increase resistance to antimicrobial compounds [46], we tested whether cheats increased the susceptibility of biofilms to antibiotics. We found that WT biofilms could resist the aminoglycoside antibiotic Tb (intercept ≠ 1; *t*_1,16_ = −0.539, *p* = 0.597; figure 3*b*), but that an increasing proportion of cheats in the biofilms led to decreased survival when challenged with Tb (*F*_1,16_ = 27.9, *p* < 0.001; figure 3*b*).

### (c) Increasing cooperation reduces the population-level consequences of cheats

**Figure 4.**
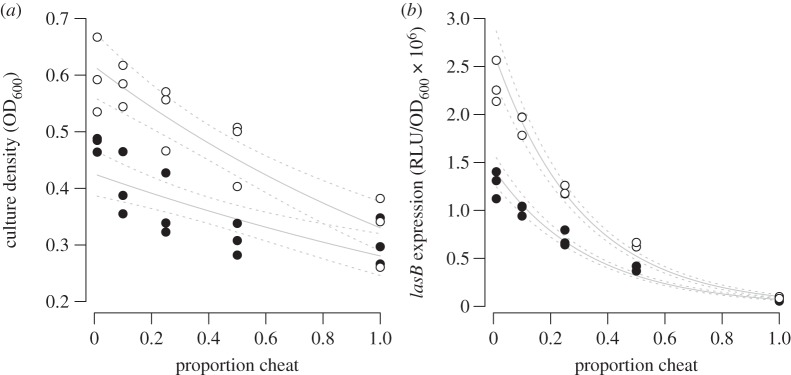
Inducing cooperation can ameliorate the population-level detriment to productivity. An increasing frequency of cheats results in a decrease in (*a*) productivity and (*b*) *lasB* expression. When the frequency of cheats is low, both of these can be partially restored by the addition of 50 µM 3-oxo-C12-HSL (open circles, signal addition; filled circles, no addition). Mixed planktonic culture consisted of the PAO1 WT and corresponding *lasR* mutant, both carrying a *lasB*::*lux* reporter gene fusion. The solid lines represent the results of linear modelling, and the dashed lines represent the 95% confidence limits placed on the linear model.

In theory, the negative consequences of cheats at the population level could be partially compensated for, if the cooperators increased their rate of cooperation [47,48]. We tested this by examining how the addition of synthetic 3-oxo-C12-HSL signal influenced the relationship between productivity and the percentage of cheats by growing the cultures planktonically in microtitre plates. We then tested that this pattern resulted from the response to added signal, by analysing QS activity using a *lasB*::*lux* fusion. We found that adding signal led to a larger increase in population productivity and *lasB* expression when the initial proportion of cheats was lower (figure 4*a,b*). Specifically, when analysing the log-transformed value of culture density and *lasB* expression, we found that the slopes of the two lines were not significantly different (culture density, *F*_3,26_ = 2.47, *p* = 0.128 and *lasB* expression *F*_3,26_ = 2.59, *p* = 0.120), but that the intercept was significantly higher when signal was added (culture density *F*_3,26_ = 39.4, *p* < 0.001 and *lasB* expression *F*_3,26_ = 89.9, *p* < 0.001).

## 4. Discussion

We determined the influence of cheats at the population level, in both planktonic and biofilm cultures. We found that: (i) a *lasR* mutant has an increased fitness in co-culture with a QS cooperating strain which is indicative of social cheating (figure 1); (ii) the thickness and density of biofilms was reduced by the presence of QS cheats (figure 2); (iii) population growth was reduced by the presence of cheats, and that this reduction was greater in biofilms than in planktonic populations (figure 3*a*); (iv) the susceptibility of biofilms to antibiotics was increased by the presence of cheats (figure 3*b*); and (v) coercing cooperator cells to increase their level of cooperation decreases the extent to which the presence of cheats reduces population productivity (figure 4).

Our results provide clear experimental support for the prediction that public good conflicts, or the problem of cooperation, can occur over cooperative behaviours in a biofilm. The presence of cheats led to a lower biofilm thickness and density (figure 2*a,b*) and an increased susceptibility to antibiotics (figure 3*b*). This negative influence of cheats occurred when we grew biofilms in both flow cells (figure 2) and microtitre plates (figure 3). Furthermore, the reduction in population growth was greater when cells were growing as a biofilm, than when they were growing in planktonic populations (figure 3*a*).

We found that when we added exogenous signal molecules to cultures, that this decreased the negative impact of cheats, but more so at lower percentages of cheats (figure 4*a*). The reason for this is that the *lasR* cheat does not respond to signal. At low percentages of cheats, there are still a high proportion of WT cooperators in the population, who will increase their cooperative production of extracellular factors, in response to the addition of signal (figure 4*b*). By contrast, at higher percentages of cheats, there are increasingly fewer WT cooperators, and so fewer cells that respond to the addition of artificial signal (figure 4*b*). This demonstrates that public good conflicts can be ameliorated by an increased level of cooperation by cooperators, but less so as cheats become more common. However, while the population level detriment to productivity can be recovered in the short term by increased cooperation, this is likely to further increase the relative fitness of a cheat, restoring the dilemma.

Many secreted factors have been shown to be important in biofilm formation and stability such as lectins, rhamnolipids and extracellular DNA and exopolysaccharides [49–51]. Many of these could potentially be exploited by non-cooperating cheats. Previous theoretical and experimental work has shown that cells which secrete extracellular polysaccharides can outcompete non-secretors in biofilms. In this case, the secreted factor is non-diffusible and not exploited by non-secretors [22,52]. Growth of biofilms in QSM is therefore likely to be multifactorial, but the production of proteases to gain access to nutrients is a major factor, and so cheating in our biofilm cultures is largely dependent upon protease exploitation (figure 1*b*). Previous work has suggested that biofilms lead to a segregation of cooperators and cheats, which reduces the ability of cheats to exploit cooperators [19,22]. It may also be that: (i) cell density (which probably varies between planktonic and biofilm populations), and (ii) conditionally regulated cooperation (such as QS) can augment the effect of biofilms on cooperation.

To conclude, as well as demonstrating how the problem of cooperation, or public goods conflict can influence microbial biofilms, our results may have clinical implications. During chronic infections, such as those found in the cystic fibrosis lung, QS *lasR* mutants are commonly isolated [53], and it is also well established that *P. aeruginosa* forms antibiotic-resistant biofilms during such infections [15]. While the basis for such tolerance is not fully understood, mutation of key QS genes renders *P. aeruginosa* biofilms more susceptible to Tb [46]. In the present paper, we have shown that as QS cheats become more common, this increases the susceptibility of biofilm populations to Tb (figure 3*b*). This could be owing simply to the increased numbers of susceptible cells (cheats) in the population or it could be owing to an increased sensitivity of all of the biofilms cells due to the formation of a poorer biofilm. In either case, the outcome is a biofilm that is easier to combat. Therefore, our results suggest that understanding the processes driving social evolution of pathogenic microbes can help explain diversity seen within clinical infections, and could even lead to novel therapeutic strategies [54].
